# Spatial and temporal regeneration patterns within gaps in the primary forests vs. secondary forests of Northeast China

**DOI:** 10.3389/fpls.2023.1305535

**Published:** 2023-11-28

**Authors:** Danni Wu, Deliang Lu, Jiaojun Zhu, Xiaowen Ge, Jinxin Zhang, Litao Lin, Xiaoyu Wang, Huaqi Liu, Guangqi Zhang

**Affiliations:** ^1^ Qingyuan Forest CERN, National Observation and Research Station, Liaoning, Shenyang, China; ^2^ CAS Key Laboratory of Forest Ecology and Management, Institute of Applied Ecology, Shenyang, China; ^3^ University of Chinese Academy of Sciences, Beijing, China; ^4^ Center for Ecological Civilization Research, Chinese Research Academy of Environmental Sciences, Beijing, China; ^5^ Jiyang College, Zhejiang A & F University, Zhuji, China; ^6^ College of Forestry, Guizhou University, Guiyang, China

**Keywords:** forest gap, natural regeneration, pattern and dynamic, primary forest, secondary forest

## Abstract

Forest gaps play an important role during forest succession in temperate forest ecosystems. However, the differences in spatial distribution and replacement patterns of woody plants (trees and shrubs) between primary and secondary forests remain unclear during the gap-filling processes, especially for temperate forests in Northeast China. We recorded 45,619 regenerated trees and shrubs in young gaps (<10 years), old gaps (10~20 years), and closed forest stands (i.e., filled gaps) in the primary broadleaved Korean pine (*Pinus koraiensis* Sieb. Rt Zucc.) forests vs. secondary forests (degraded from primary forests). The gap-filling processes along horizontal (Cartesian coordinate system) and vertical (lower layer: 0~5 m, medium layer: 5~10 m, and upper layer: >10 m) dimensions were quantified by shade tolerance groups of trees and shrubs. We found that gap age, competition between species, and pre-existing regeneration status resulted in different species replacement patterns within gaps in primary vs. secondary forests. Gap formation in both primary and secondary forests increased species richness, with 33, 38, 39, and 41 in the primary closed stands, primary forest gaps, secondary closed stands, and secondary forest gaps, respectively. However, only 35.9% of species in primary forest gaps and 34.1% in secondary forest gaps successfully reached the upper layer. Based on the importance values (IVs) of tree species across different canopy heights, light-demanding trees in the upper layer of the secondary forests were gradually replaced by intermediate and shade-tolerant trees. In the primary forests, Korean pine exhibited intermittent growth patterns at different canopy heights, while it had continuous regeneration along vertical height gradients in the secondary forests. The differences in Korean pine regeneration between the primary and secondary forests existed before gap formation and continued during the gap-filling processes. The interspecific competition among different tree species gradually decreased with increasing vertical height, and compared to the primary forests, the secondary forests showed an earlier occurrence of competition exclusion within gaps. Our findings revealed the species replacement patterns within gaps and provided a further understanding of the competition dynamics among tree species during the gap-filling processes.

## 1 Introduction

The escalating issue of forest degradation has emerged as a formidable global challenge confronting humanity (Global Forest Resources Assessment, 2020). How to guide degraded forests toward positive succession has become a global concern (Hua et al., 2022). Degraded forests, such as secondary forests, often require forest management to accelerate their restoration to the primary forest with more stability and resilience. Strategies like near-natural forest management, which imitates forest gaps, have been proposed to enhance species diversity and guide positive succession in secondary forests (Jacquemyn et al., 2003; Wang et al., 2021; Lu et al., 2023). Forest gaps, caused mainly by strong winds, lightning storms, or artificial disturbances, have the potential to alter the above- and below-ground resources in closed forests (McClure and Lee, 1993; Jacquemyn et al., 2003; Seidl et al., 2017), thereby affecting the structural and compositional progression of the forest (Hale, 2003; Seidl et al., 2017).

The intermediate disturbance hypothesis suggested that disturbances, such as forest gaps, promote the coexistence of species with different life histories (Hubbell et al., 1999; Lara-Romero et al., 2016). The spatial distribution pattern of tree species in canopy gaps represents the competition they are experiencing within the gaps and can also help researchers predict the future development trajectory of canopy gaps (Jacquemyn et al., 2003; Owen et al., 2017; Wang et al., 2017; Omelko et al., 2018; Wang et al., 2021). Tree species with different shade tolerance will survive in different positions within the forest canopy (Lu et al., 2018). For example, Poznanovic et al. (2014) observed that intermediate shade-tolerant *Betula alleghaniensis* was mainly located in the south part of the nine-year-old large gaps. Meanwhile, light-demanding *P. tabulaeformis* preferred regenerating in the northeastern part of the gap created seven years ago (Wang et al., 2017). Understanding the spatial distribution patterns of different species within forest gaps can provide important clues regarding the future development trends of the forest and serve as a scientific basis for ecological conservation and management (Agyeman et al., 1999; Thom and Seidl, 2016).

Moreover, the resource competition theory suggests that the horizontally and vertically spatial distribution patterns of species are also determined by resource competition (Larocque et al., 2013; Nakashizuka and Matsumoto, 2002). The types of competition experienced by individual trees vary at different vertical heights within canopy gaps (Dai et al., 2018). In the lower layers of canopy gaps, shrubs often compete for growing space and negatively impact the growth of tree seedlings (Kern et al., 2012). In the middle and upper layers of canopy gaps, the competition between tree species gradually decreases, and the spatial distribution pattern of trees is controlled by resource heterogeneity at the landscape scale (Raymond et al., 2006; Wang et al., 2021). Only a few tree species can successfully grow from lower to middle and upper layers (Zhu et al., 2021). The average tree species regeneration from a horizontal perspective may lead to a limited estimation of the progress of tree species succession in forest gaps (Lu et al., 2023). The vertical arrangement of temperate forests holds significant importance once the trees have been established (Nakashizuka and Matsumoto, 2002). According to Hao et al. (2007), the vertical structure plays a crucial role in the regeneration of populations and the dynamics of communities. It also provides valuable insights into population dynamics and competition in different height layers. Therefore, it is necessary to have a clear understanding of the horizontal and vertical regeneration processes of different shade-tolerant tree species within forest gaps (Martin et al., 2021; Lu et al., 2023).

Secondary forests formed due to severe disturbances in primary forests account for over 70% of the forests in Northeast China (Zhu et al., 2021). Unlike primary forests, secondary forests have the characteristics of destruction or loss of basic structure and inherent functions (Finegan, 1996), decreased biodiversity (Turner et al., 1997), weakened stability and resilience (Finegan, 1996), and low system productivity (Guariguata and Ostertag, 2001). For example, Zhang et al. (2009) observed that the Shannon diversity of the primary broadleaved Korean pine forest was higher than that in the secondary forest. Previous studies indicated that it would take over 200 years for secondary forests to restore functions similar to primary forests under natural development (Wang et al., 1993). The current issue for forest managers is how to guide the positive succession of secondary forests to restore the forest structure to resemble that of the primary forests. In recent decades, forest managers have developed various forest management programs, especially simulating natural gap disturbances, to accelerate the restoration of degraded forests and enhance ecosystem services (Kern et al., 2017). Consequently, understanding the process and mechanisms of gap regeneration is important to achieve the management goals of promoting natural forest succession (McClure and Lee, 1993; Raymond et al., 2006).

This study aims to compare the horizontal and vertical regeneration patterns of tree species with different shade tolerances (i.e., light-demanding, intermediate, and shade-tolerant) in the primary and secondary forests during the gap-filling processes. We used point pattern analysis methods to explore the spatial distribution patterns of trees and shrubs at different vertical layers and the underlying implications for competition. We investigated all trees and shrubs within 12 natural gaps in the primary and secondary forests. The specific questions were: (1) How does the regeneration of tree species change along vertical dimensions during gap-filling in primary vs. secondary forests? (2) How do tree species of different shade tolerance fill the gaps regarding distribution patterns at different vertical layers? (3) How does the interspecific competition change at different vertical layers in primary vs. secondary forest gaps? We hypothesized that: (1) As the gap closure process progresses, the IV of trees in both forest types will increase, but species-specific differences exist. (2) Light-demanding species dominate in the early stages of gap formation, while intermediate and shade-tolerant species gradually replace part of the light-demanding species. (3) Compared to primary forests, competition for trees in secondary forests might be less intense.

## 2 Materials and methods

### 2.1 Study area

This study was carried out within the Lushuihe Forest Bureau, situated in the northwestern region of the Changbai Mountains (127°29’-128°02’ E, 42°20’-42°40’ N). The study area is characterized by mixed broadleaved Korean pine forests, and it experiences a typical temperate continental climate. The region has an average annual temperature of 2.8°C and an annual precipitation ranging from 800 to 1040 mm. The elevation in this region ranges from 600 to 800 meters above mean sea level, and the soil is typical mountainous dark brown forest soil (Zhao et al., 2013). Before experiencing significant disturbances, the forest type in the Lushuihe Forest Bureau was the primary broadleaved Korean pine forest. The dominant species were Korean pine and broadleaved trees such as *Tilia amurensis*, *Acer mono*, *Fraxinus mandshurica*, and *Juglans mandshurica* (Zhao et al., 2013). The secondary forest in our study area is derived from natural regeneration after clear-cutting and is dominated by *Betula platyphylla*. In 1998, the Chinese government initiated implementing Natural Forest Conservation Projects, and the Lushuihe Forestry Bureau began prohibiting timber logging (Yu et al., 2019).

### 2.2 Gap selection

We set up six treatments to compare the gap regeneration in different development stages, including primary young gap (PYG), primary old gap (POG), primary closed stand (PCK), secondary young gap (SYG), secondary old gap (SOG) and secondary closed stand (SCK). Each treatment was repeated three times.

We adopted the belt transect method in the field investigation in 2019. A total of 198 and 30 forest gaps were pre-selected in the primary broadleaved Korean pine forest and in the secondary birch forest, respectively. The purpose of our pre-selection was to identify ideal forest gaps (and CK plots). For all pre-selected gaps, we measured gap size, recorded gap shape, and estimated gap age using tree ring analysis, and then we decided which gap could be used. The minimum distance between any two sample plots was more than 100 m, and there were no other gaps between any two selected plots. Then, we only selected forest gaps that meet the following conditions for field surveys: (1) The diameter-to-height ratio of forest gaps >0.73 (medium gap and large gap); (2) No obvious signs of human or animal disturbances; (3) The tree core shows obvious growth and release, which can accurately determine the age of forest gaps (Rozas, 2003). Consequently, six medium and large gaps were selected from the primary broadleaved Korean pine and secondary forests. Three gaps in the primary forest were 440.7 ± 153.7 m^2^ on average with a gap age of 6.3 ± 1.7 years as the PYG, and three gaps were 612.7 ± 115.2 m^2^ on average with a gap age of 15 ± 1.6 years old as the POG. Three gaps in the secondary forest were 395.6 ± 80.7 m^2^ on average with a gap age of 6 ± 1.4 years old as the SYG, and three gaps were 417.6 ± 78.6 m^2^ on average with a gap age of 14 ± 2.2 years old as the SOG. We selected three control plots (30 m × 30 m) in PCK and SCK as the plot filled and closed through forest gaps.

### 2.3 Gap regeneration surveys

We recorded a total of 45,196 trees and shrubs with height >0.1 m in the plots, documenting all their species, coordinates, heights, diameters at breast height (DBH) or root collar diameter (RCD), and other relevant indicators. We divided them into three height groups, including the lower layer (height: 0~5 m), medium layer (height: 5~10 m), and upper layer (height: >10 m) within the PYG, POG, PCK, SYG, SOG, and SCK plots. We established a coordinate system with the center of the forest gap as the origin (0, 0). The north-south direction of the forest gap is the Y-axis, and the east-west direction of the forest gap is the X-axis. To map the regeneration locations more accurately, we divided every plot into 100 subplots (3 m × 3 m) to avoid missing individuals during the investigation. An ultrasound-based positioning instrument (Fieldscout TDR 350, Haglöf PosTex Långsele, Sweden) was used to acquire the precise coordinates of each individual, which could improve the accuracy of spatial point pattern analysis results.

### 2.4 Statistical analysis

#### 2.4.1 Regeneration within gaps

To assess the transition patterns of tree species in different stages of gap closure between primary and secondary forests, we calculated the importance values of different tree species in PYG, POG, PCK, SYG, SOG, and SCK. In order to explore the regeneration dynamics of different tree species at varying vertical heights during different stages of gap closure in primary and secondary forests, we computed the importance values of different tree species in lower layer (height: 0~5 m), medium layer (height: 5~10 m) and upper layer (height: >10 m) within the PYG, POG, PCK, SYG, SOG, and SCK plots. The importance value was calculated with the following equations:


$$IV=\frac{RD+RF+RC}{3}$$

$$IV=\frac{\frac{n_{i}}{\sum_{i=1}^{s}n_{i}}*100+\frac{a_{i}}{\sum_{i=1}^{s}a_{i}}*100+\frac{f_{i}}{\sum_{i=1}^{s}f_{i}}*100}{3}$$

where *RD* is the relative dominance, *RF* is the relative frequency, *RC* is the relative coverage, *n_i_* is the number of individuals of the *i*th species, *a_i_* is the basal area belonging to the *i*th species, *f_i_* is the number of quadrats the *i*th species appeared, and *S* is the total number of species.

To assess the regeneration process in gaps of different vertical layers, we calculated the species richness in the lower layer (height: 0~5 m), medium layer (height: 5~10 m), and upper layer (height: >10 m) of PYG, POG, PCK, SYG, SOG, and SCK plots. The species richness was calculated as the number of species in each plot.

To assess the regeneration process in gaps of different ages, we calculated the Shannon diversity index of PYG, POG, PCK, SYG, SOG, and SCK plots. The Shannon diversity index was calculated with the following equations:


$$H=-\sum_{i=1}^{s}\left(Pi\right)\left(\text{ln}Pi\right)$$

where *Pi* is the proportion of the entire species community, and *S* is the total number of species in plots.

To assess the regeneration growth in gaps of different ages, we analyzed the height differences of light-demanding, intermediate, and shade-tolerant species in PYG, POG, PCK, SYG, SOG, and SCK plots. One-way ANOVA was used to test the effects of gap age on the heights of woody species. Tukey’s *post hoc* tests were used to test the differences between different gap ages using SPSS (SPSS software, 22nd edition, Chicago, USA).

#### 2.4.2 Univariate and bivariate spatial patterns

Point pattern analysis is widely applied in describing gap regeneration patterns (Poznanovic et al., 2014; Wang et al., 2017). The univariate spatial patterns of trees at the lower layer (height: 0~5 m), medium layer (height: 5~10 m), and upper layer (height: >10 m) in PYG, POG, PCK, SYG, SOG, and SCK plots were analyzed using *g(r)*. Point pattern analysis was conducted using the Programita software (Wiegand et al., 2016).

Many previous studies used *K(r)* to calculate the spatial pattern, but *K(r)* has a nature of cumulation (Ripley, 1977). The pair correlation function *g(r)* is widely used because of its non-cumulative character. The pair correlation function employs annuli as distance categories rather than circles. *g(r)* is related to the *K(r)* (Ripley, 1977) as follows:


$$K\left(r\right)=\frac{s}{n\left(n-1\right)}\sum_{m}\sum_{n}I\left(d_{mn}\leq r\right)e_{mn}$$

$$g\left(r\right)=\frac{d}{dr}K\left(r\right)/\left(2\pi r\right)$$

where *s* is the area of the forest gaps, *n* is the number of points, *m* and *n* are all ordered point pairs, *I* is an indicator (1 if *d_mn_* ≤ 1), *d_mn_* is the distance between two points, and e is an edge correction. Points above the upper *g(r)* envelope represent clustered, and points below the *g(r)* envelope represent regularity. The *g(r)* envelopes were derived from 199 Monte Carlo simulations of CSR (Wiegand et al., 2016).

The bivariate relationship between trees and shrubs in PYG, POG, PCK, SYG, SOG, and SCK plots was analyzed by *g_12_(r)*:


$$g_{12}\left(r\right)=\frac{d}{dr}K_{12}\left(r\right)/\left(2\pi r\right)$$

If the observed *g_12_(r)* was located above or below the simulation envelope at a specific distance r, indicating the attraction or repulsed pattern of shrubs and trees, respectively. If the observed *g_12_(r)* was located in the simulation envelope, indicating the independent relation of shrubs and trees. In the context of point pattern analysis, the significance level of 199 Monte-Carlo test was 0.02.

#### 2.4.3 Kernal density estimation for density

Kernel density estimation (KDE) was used to analyze the density distribution of light-demanding, intermediate, and shade-tolerant trees in lower, medium, and upper layers of PYG, POG, PCK, SYG, SOG, and SCK (Langford and Unwin, 1994).

The KDE analysis, introduced by Bowman (1985), is a nonparametric statistical method. The KDE analysis utilizes the quadratic kernel function to represent a smooth surface for every data point. The KDE analysis enables the computation of the magnitude per unit area, as described by Silverman, 1986. The kernel density estimator is defined as follows:


$$f_{r}\left(x\right)=\frac{1}{pr}\sum_{i=1}^{n}S\left(\frac{x-x_{i}}{r}\right)$$

where *S* is a non-negative function with an integral of 1, with an average value of zero, *p* is the number of the points of trees and shrubs *(x)*, and *r* is the search radius (Silverman, 1986). The research radius r is 1 m in our research. We converted the initial coordinates of the trees and shrubs into a new coordinate system, with the origin coordinates set at the center of each gap. These new coordinates system effectively represented all 45,196 trees and shrubs in the plots, with the forest gap center points as a common anchor point. Trees and shrubs were first grouped by layers (lower layer, medium, and upper layer) and shade-tolerance (light-demanding, intermediate, and shade-tolerant) and were aligned based on the coordinates of the center of each gap. Then, we ran kernel density estimates for the 12 gaps and 6 closed forest stands. ArcMap 10.1 (ESRI, 2012) was used to calculate the kernel density estimates for PYG, POG, PCK, SYG, SOG, and SCK.

## 3 Results

### 3.1 General regeneration patterns

**Figure 1:**
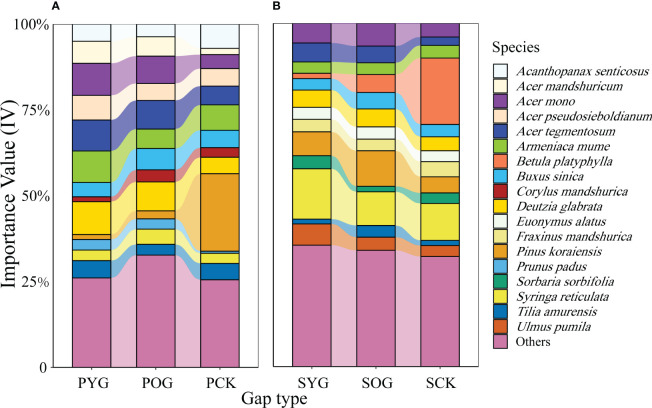
Importance value (IV) of all species in **(A)** primary and **(B)** secondary forest. PYG, primary young gap; POG, primary old gap; PCK, primary closed forest; SYG, secondary young gap; SOG, secondary old gap; SCK, secondary closed forest.

Generally, the number of species in the PCK, the primary forest gap (PYG and POG), SCK, and the secondary forest gap (SYG and SOG) were 33, 39, 38, and 41, respectively (**Table S2**). The Shannon diversity index of PCK, PYG, POG, SCK, SYG, and SOG were 2.50, 2.41, 2.75, 2.54, 2.08 and 2.67, respectively. However, the IV changes in tree species in the primary and secondary forests were quite different (**Table S1**). According to the data in **Figure 1**, the IV of Korean pine in the primary forest gap was far lower than that in the secondary forest gap, which was 1.4% in PYG, 2.3% in POG, 6.9% in SYG and 10.4% in SOG, respectively. *Acer* spp. maintained high IV in the gap plots and control plots of the primary forest or secondary forest (**Figure 1**).

### 3.2 Regeneration pattern in different layers

**Figure 2:**
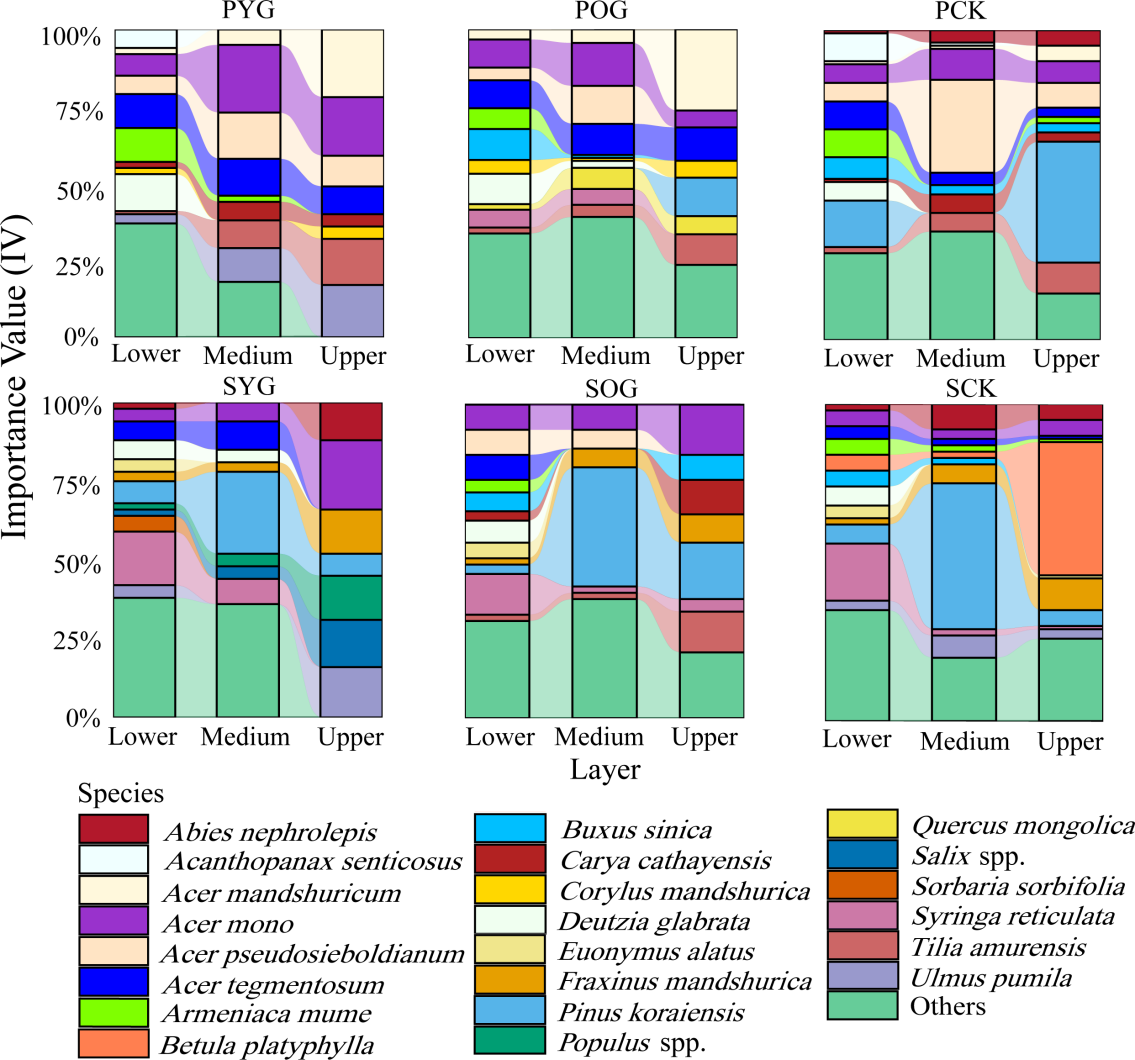
Importance value (IV) of all species in lower layer (0~5 m), medium layer (5~10 m), and upper layer (>10 m) in primary and secondary forest. PYG, primary young gap; POG, primary old gap; PCK, primary closed forest; SYG, secondary young gap; SOG, secondary old gap; SCK, secondary closed forest.

In both primary and secondary forest gaps, there was a trend of decreasing species richness with increasing vertical height (**Figure 2**). However, the IV of some species in primary and secondary forests showed a distinct pattern across lower, medium, and upper layers. As was apparent from **Figure 2**, Korean pine had the highest importance value in the medium layer of secondary forest gaps but barely had any regeneration in primary forest gaps. *Acer* spp. had a high proportion in three layers of primary and secondary forests. Surprisingly, the importance value of *B. platyphylla* in the upper layer of the SCK was 50%, while its importance value in the lower and medium layers of the SCK tended to be zero. Similar results were also found in the primary forest.

**Figure 3:**
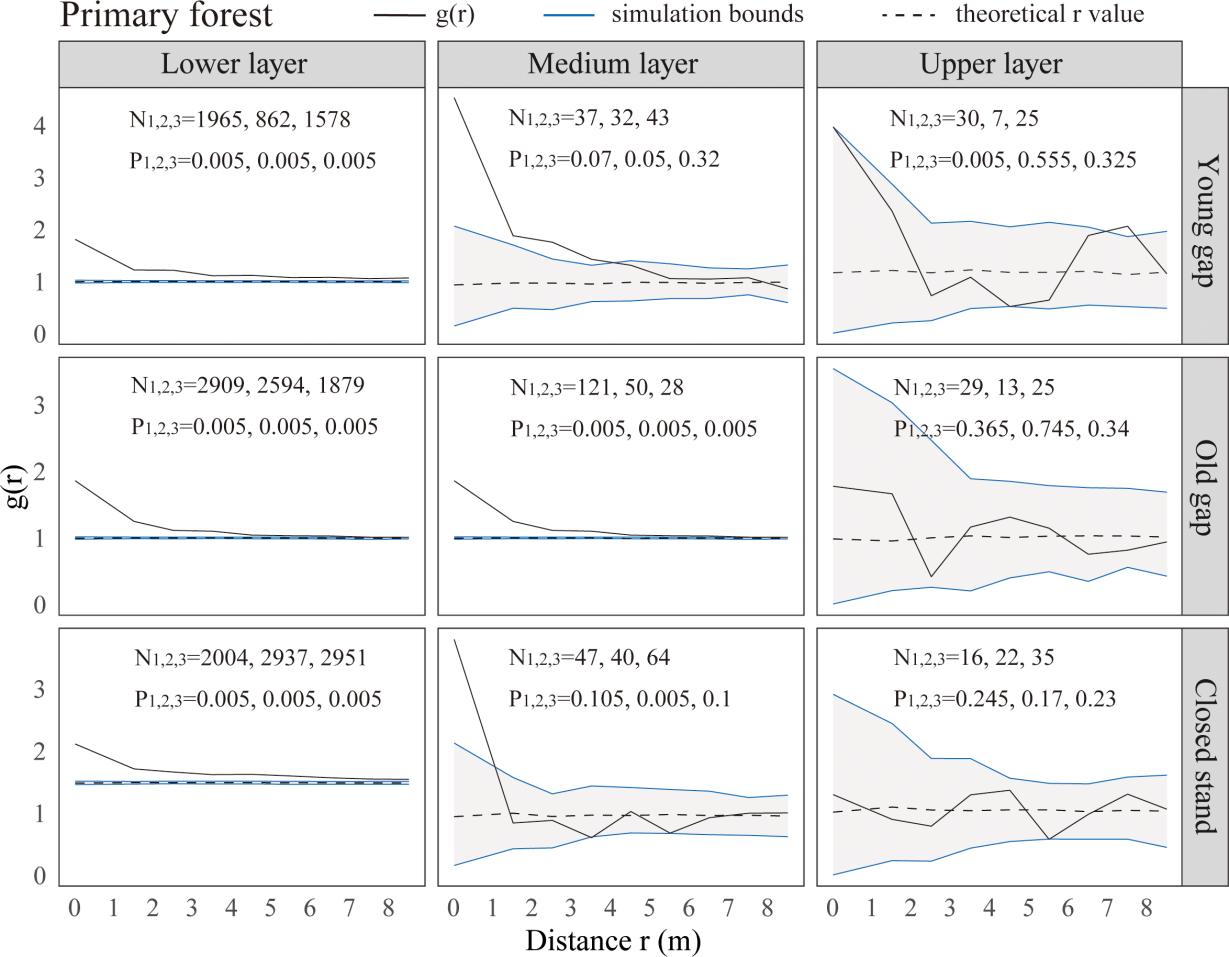
Univariate spatial pattern analysis of all trees in the lower (0~5 m), medium (5~10 m), and upper layer (>10 m) in primary forests. Each point pattern analysis was repeated three times, with N representing the number of trees and P representing the difference between simulation values and observed values, g(r) function for 199 Monte Carlo simulations of the null model with a 98% confidence envelope for a completely random point process.

**Figure 4:**
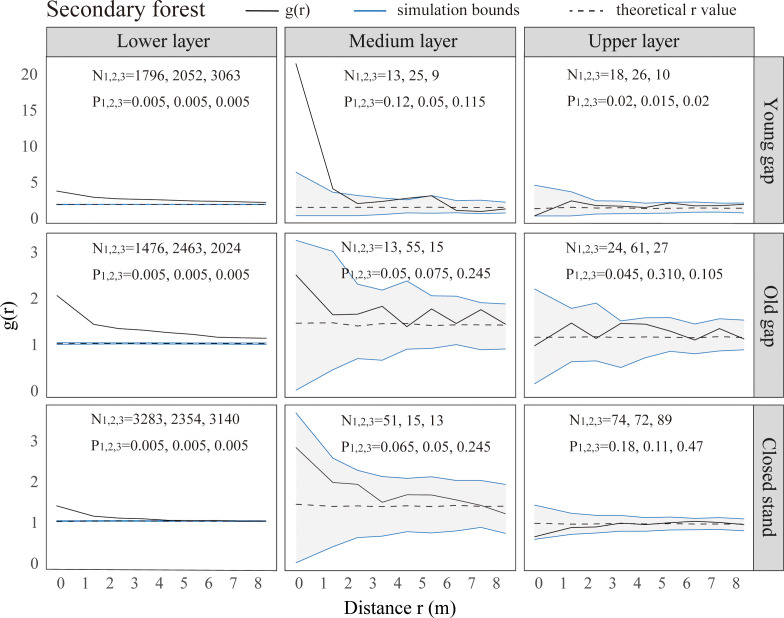
Univariate spatial pattern analysis of all trees in the lower (0~5 m), medium (5~10 m), and upper layer (>10 m) in secondary forests. Each point pattern analysis was repeated three times, with N representing the number of trees and P representing the difference between simulation values and observed values, g(r) function for 199 Monte Carlo simulations of the null model with a 98% confidence envelope for a completely random point process.

**Figure 5:**
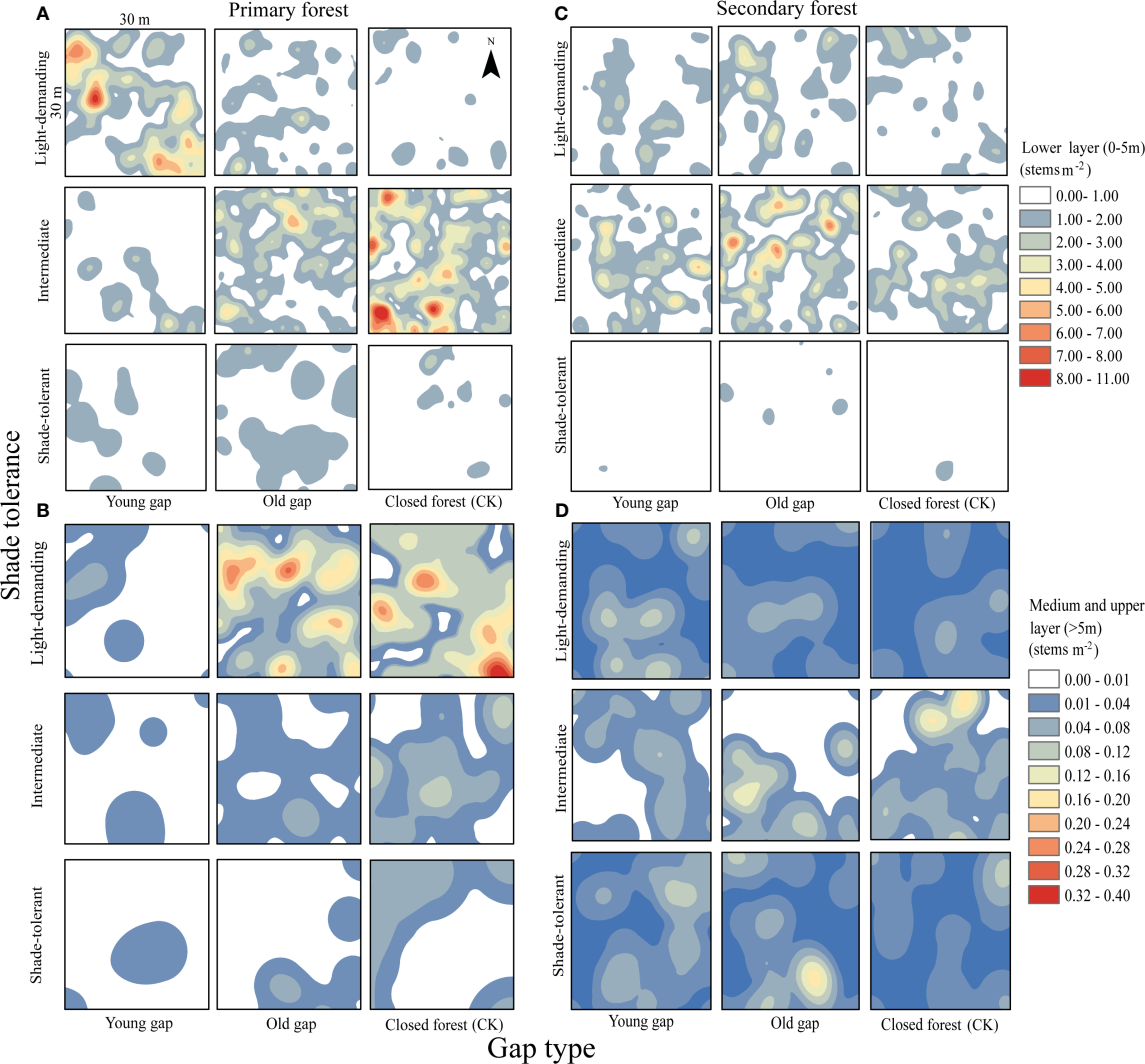
Mean kernel stem density estimates for light-demanding, intermediate, and shade-tolerant species in the lower (0~5 m), medium (5~10 m), and upper layer (>10 m). PYG, primary young gap; POG, primary old gap; PCK, primary closed forest; SYG, secondary young gap; SOG, secondary old gap; SCK, secondary closed forest. **(A)** lower layer in primary forest, **(B)** medium and upper layer in primary forest, **(C)** lower layer in secondary forest, **(D)** medium and upper layer in secondary forest.

**Figure 6:**
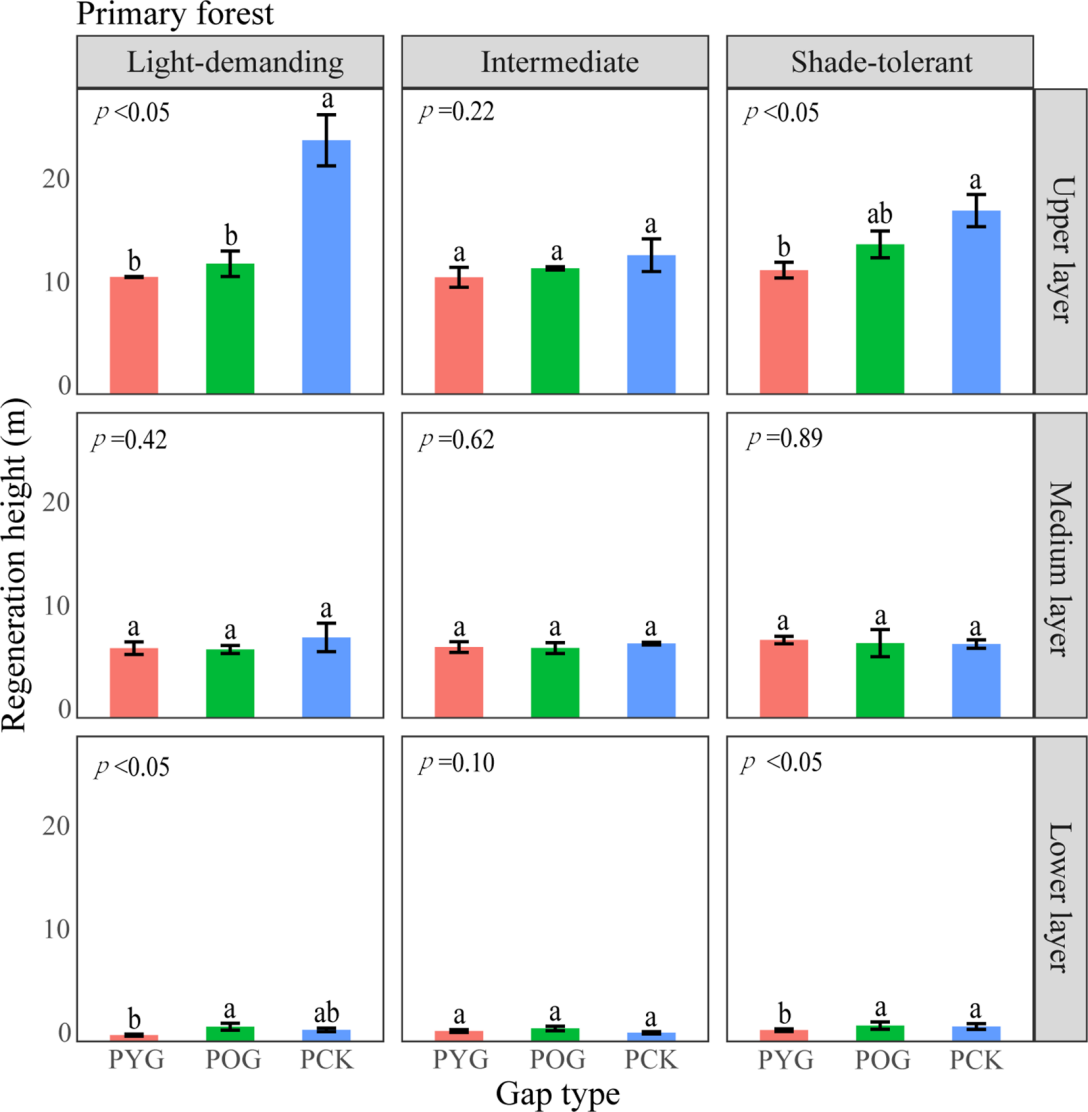
A one-way ANOVA of the height of trees with different shade tolerance (light-demanding, intermediate, and shade-tolerant) in primary forests. PYG, primary young gap; POG, primary old gap; PCK, primary closed forest. Different letters represented significant differences at the *p*<0.05. Error bars represented standard deviation.

**Figure 7:**
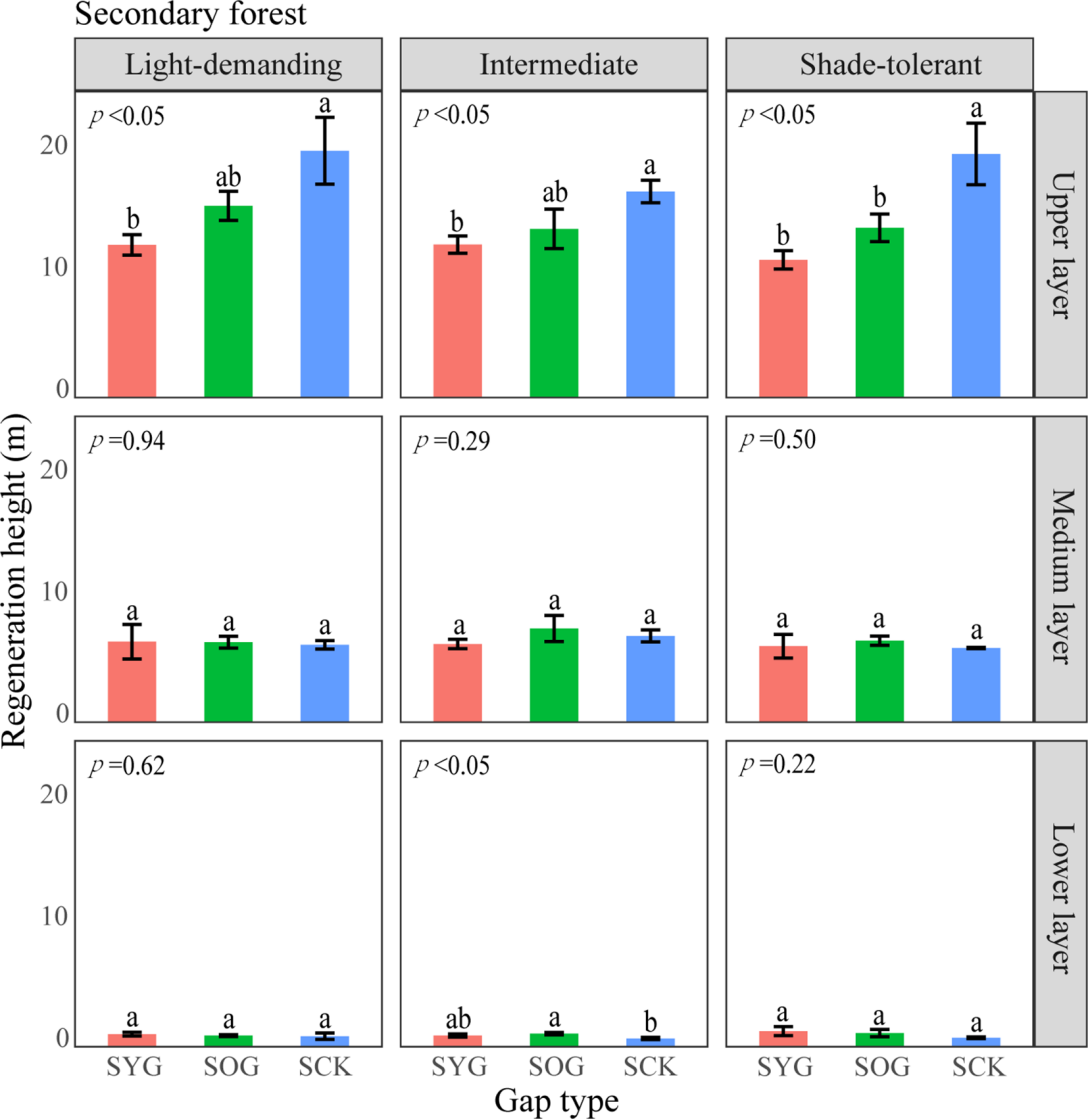
A one-way ANOVA of the height of trees with different shade tolerance (light-demanding, intermediate, and shade-tolerant) in secondary forests. SYG, secondary young gap; SOG, secondary old gap; SCK, secondary closed forest. Different letters represented significant differences at the *p*<0.05. Error bars represented standard deviation.

**Figure 8:**
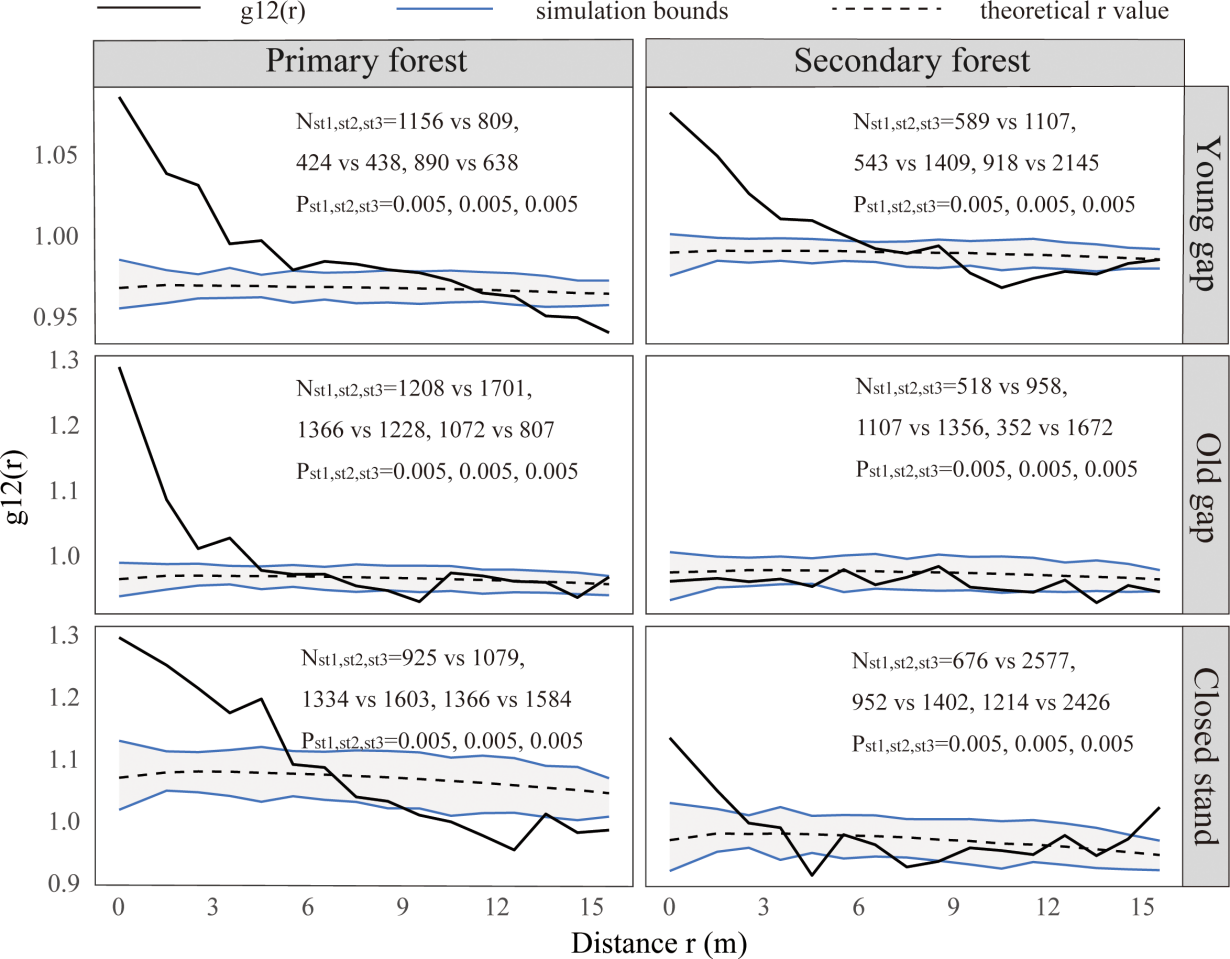
Bivariate spatial patterns for shrubs and trees in the lower (0~5 m) layer. PYG, primary young gap; POG, primary old gap; PCK, primary closed forest; SYG, secondary young gap; SOG, secondary old gap; SCK, secondary closed forest. Each point pattern analysis was repeated three times, with N_st_ representing the number of shrubs and trees and P representing the difference between simulation values and observed values, g_12_(r) function for 199 Monte Carlo simulations of the null model with the 98% confidence envelope for a complete independence pattern.

From the pattern analysis results in **Figures 3**, **4**, it can be seen that trees tended to randomly distribute as the height layer increased. Trees in the upper layer were spatially random across all scales (100%) examined in PYG, POG, PCK, SYG, SOG, and SCK (**Figures 3**, **4**). Similarly, trees in the lower layer were clustered at all distances (100%) examined in PYG, POG, PCK, SYG, and SOG, and 50% scale in SCK. However, the trees in the medium layer clustered in PYG, POG, and PCK at a small scale (**Figure 3**), contrasting with a random distribution at 100% distances in the medium layers of SOG and SCK (**Figure 4**). The density distribution of light-demanding and intermediate trees was denser than shade-tolerant trees (**Figure 5**). As the process of gap closure advanced, there was a gradual decline in the highest density of light-demanding trees in the lower layer appealing, with a discernible pattern of decreasing from 8-11 stems m^-2^ in the PYG to 3-4 stems m^-2^ in the POG, and ultimately reaching 1-2 stems m^-2^ in the PCK (**Figure 5**). However, the density of shade-tolerant trees in the lower layer of secondary forests remains relatively stable (**Figure 5**). In both primary and secondary forest gaps, there was a similar pattern of the density of intermediate trees increasing with the age of the gaps in the lower layer regeneration (**Figure 5**). The height of upper layer trees increased gradually with the gap closure of primary and secondary forests (**Figures 6**, **7**). However, the average height of light-demanding, intermediate, and shade-tolerant trees remained stable in the medium layer of primary forest and secondary forest (**Figures 6**, **7**). Competition between trees and shrubs in the lower layer showed a small-scale positive correlation and a large-scale negative correlation in PYG, SYG, and PCK (**Figure 8**). The mutual exclusion between trees and shrubs in primary forests intensified with forest succession. Compared with secondary forests, the competition relationship between trees and shrubs was more obvious in primary forests (**Figure 8**).

## 4 Discussion

### 4.1 General regeneration patterns

We found that forest gaps increased species richness in both primary and secondary forests. Gap formation increases light penetration, creating opportunities for plants to thrive and for new seedlings to establish (Abd Latif and Blackburn, 2010). This led to a greater variety of habitats and resources, ultimately supporting a more comprehensive range of species in the forest ecosystem (Thom and Seidl, 2016; Lu et al., 2023). In accordance with the findings of Liu et al. (2003), our study also observed a similar pattern of Shannon diversity index in primary and secondary forests, namely: PYG<PCK<POG and SYG<SCK<SOG. This pattern can be explained by the early stages of forest gap formation, during which dominant species had more regeneration compared to other species, resulting in a lower Shannon diversity index in young gaps. As the forest gaps aged, the diversity of tree species increased, leading to a rise in the Shannon diversity index (Liu et al., 2003). Our results showed that the variation in importance values of the same tree species differs between plots in the primary and secondary forests. The importance value of Korean pine was much lower in the primary forest gaps than in the secondary forest gaps. To investigate the reasons, we inferred the advanced regeneration of Korean pine before gap formation based on the calculated age of the Korean pine (Ge et al., 2022). The advantage of Korean pine regeneration in the secondary forest was already present before gap formation (**Figure S1**). After gap formation, the regenerating Korean pine maintained this advantage (Liu and Li, 1987). Previous results showed a positive correlation between the growth of Korean pine seedlings and canopy transparency (Zhang et al., 2013). The light intensity in the secondary forest gap was higher than in the primary forest gap, which led to the good regeneration of Korean pine in the secondary forest gap. Fan and Xu (2019) claimed that habitat filtering and intraspecific density constraints simultaneously affect seedling survival. The other reason for the poor regeneration of Korean pine in the primary forest might be the high proportion of Korean pine within the primary broadleaved Korean pine forest, resulting in the conspecific negative density dependence phenomenon (Fan et al., 2017; LaManna et al., 2020).

Conversely, the importance value of *Acer* spp. was notably high in the forest gaps and closed stands of the primary forest, and a similar trend was observed in the secondary forest. (Ye et al., 2014). Some studies reported that the regeneration of *Acer* spp. was not sensitive to canopy disturbances (Yu and Hao, 1998), which was consistent with our results. Although *Acer* spp. were not sensitive to the formation of canopy gaps in both primary and secondary forests, they had high IVs in all plots. This suggested that *Acer* spp. might become the main tree species in the mixed broadleaved Korean pine forest.

### 4.2 Regeneration pattern in different layers

The proportion of importance values of Korean pine at different height layers in the primary forest plots differed from those at different height layers in the secondary forest plots. It was consistent that the Korean pine’s medium layers (Li and Wang, 1986) were barely absent in the primary forest gap and closed stand (Zhang et al., 2022). In our research, the importance value of Korean pine was relatively high in the medium layer of secondary forests but relatively low in the lower and upper layers. This meant Korean pine continuously filled gaps from the lower layer to the medium layer and then to the upper layer in secondary forests. The species richness of shrubs and trees in the understory of secondary forests was high. As the fill-in process of the medium and large forest gaps, only several tree species could successfully ascend from the lower layer to the medium layer and then extend from the medium layer to the upper layer, ultimately completing the filling process of the canopy. Our results showed that the IV of shade-tolerant trees in the upper layers of the SCK (1.7%) was far lower than that of shade-tolerant trees in the upper layers of the PCK (14.8%). In the lower layer of the primary forest gap, there was a decline in the density of light-demanding trees as the age of the forest gap increased. Conversely, there was an increase in the density of intermediate and shade-tolerant trees. Similar to our research findings, in canopy gaps within temperate hardwood forests in Connecticut, USA, there was also an observed phenomenon where light-demanding species initially dominated but were eventually replaced by shade-tolerant species (Martin et al., 2021). This phenomenon was likely caused by the suitable light conditions after gap formation, promoting the growth of light-demanding species, which subsequently made the light conditions more favorable for the growth of shade-tolerant tree species (Martin et al., 2021). However, there were also studies suggesting that the differences in growth rates between tree species with varying light requirements were insufficient in the short term to result in the complete replacement of one species by another (Guldin and Lorimer, 1985; Liptzin and Ashton, 1999). Therefore, based on the current trend of species importance value changes, we predicted that the proportion of shade-tolerant trees in the upper layer would increase with forest succession.

The formation of gaps had expanded the scale of tree aggregation, with the aggregation scale of medium layers ranging from 0-1m in PCK,0-4 m in PYG, and 0-6 m in POG. Given these variations in scale, it is necessary to focus on the competitive individuals of the dominant tree species within these ranges and promptly implement forest management practices (Wang et al., 2017). Ripley (1977) suggested that an aggregated distribution pattern implied that individual trees were still engaged in intense competition, while a random distribution pattern was the consequence of community-level competition. Our research findings suggested that there was still competition among trees with different scales in the medium layer of primary forest gaps. The intensity of this competition was greater within old forest gaps compared to young forest gaps and control plots. In the medium layer of older gaps and closed stands of secondary forests, trees had completed their competition and exhibited a random distribution pattern. Some studies suggested that the spatial pattern of trees was related to the gap size. For example, Wang et al. (2017) indicated that as the gap size increased (from 49.3 m^2^ to 185.8 m^2^), the aggregation of Chinese pine in forest gaps increased. Other researchers argued that the aggregated distribution was due to heterogeneity within the study area or the dispersal patterns of seeds (Grubb, 1977; Hubbell, 2001). Our findings indicated that the gap formation in both primary and secondary forests did not affect the clustered distribution in the lower layer and random distribution in the upper layer. The likely reason for the difference in distribution patterns in the medium layer of primary and secondary forest gaps was that the growing conditions in secondary forests were more suitable for tree growth, while the resources within primary forest gaps were limited.

The differences in height and density distribution across vertical layers also indicated differences in the primary and secondary forest regeneration processes (Tesfaye et al., 2010). The average height of shade-tolerant trees in the lower layer of the POG was significantly higher than that in the PYG and PCK. With the gap-filling progress, the density distribution of light-demanding trees gradually decreased in the lower layer. Similarly, the average height of intermediate trees in the lower layer of SOG was significantly higher than that in the SYG and SCK. However, the density distribution of intermediate trees in the lower layer remained unchanged. These trends indicated that light-demanding tree species were more likely to access the medium layer at an earlier stage within the lower layer of primary forest gaps. Theoretically, differences in tree height growth contributed to niche differentiation, ultimately allowing for the coexistence of multiple species (Dekker et al., 2009). Lu et al. (2023) observed a significant decrease in the density of light-demanding species in the lower layer, suggesting a lack of new members to complement the population. Consequently, it can be inferred that light-demanding species may only have a single opportunity for success, but intermediate species maintain continuous renewal in forest gaps.

### 4.3 Competition between trees and shrubs

Our findings indicated that the correlation between shrubs and trees varied with scale, exhibiting a trend of positive correlation at small scales in PYG, POG, PCK, SYG, SOG, and SCK and negative correlation at large scales in PYG and PCK. Previous research showed that the correlation pattern at small scales was attributed to biological characteristics (Holmgren et al., 2015). Our research indicated that the formation of forest gaps did not change the positive or independent relationship between shrubs and trees on a small scale (0-4 m). Some studies also suggested that the presence of shrubs promoted the regeneration of trees (Holmgren et al., 2015; Urza et al., 2019). Shrubs could relieve non-biological stress by reducing solar radiation, wind speed, and soil temperature, thereby reducing water loss caused by plant transpiration and soil moisture loss caused by evaporation (Lortie and Cushman, 2007). Shrubs could also inhibit tree growth by blocking moss’s direct inhibitory effect on them (Limpens et al., 2014). In addition, shrubs could indirectly promote the growth of tree seedlings by protecting them from herbivorous animals (Kern et al., 2012). However, the negative correlation between trees and shrubs at a large scale in the primary forest plots was opposite to the positive association at a large scale in the secondary forest plots. This could be related to the differences in environmental conditions within the primary and secondary forests. The correlation effect at large scales was due to environmental factors (Holmgren et al., 2015). Beckage et al. (2000) suggested that the high amount of shrub regeneration directly offsets the quality of the regeneration environment provided by forest gaps. Removing shrubs from primary and secondary forests could promote seedling growth of shade-tolerant trees (Yin et al., 2023). Once a seedling’s height growth surpasses the shrub layer, it may have a higher chance of successfully entering the canopy layer, as there are fewer potential competitors in the forest gap. Understory shrubs reduced seedling recruitment by shading out light (Kern et al., 2012). Therefore, in both primary and secondary forest plots, shrubs protected tree regeneration at small scales. However, at large scales, shrubs competed with trees for growth resources in newly formed forest gaps.

## 5 Conclusions

In this study, we used point pattern analysis to compare the gap-filling processes of tree species in primary and secondary forest gaps across horizontal and vertical dimensions. We found that the formation of canopy gaps in both primary forests and secondary forests indeed increased tree species richness. However, the main tree species in the primary broad-leaved Korean pine forest, such as *Quercus mongolica*, *F. mandshurica*, and *T. amurensis*, have not regenerated well in the lower layer. *Q. mongolica*, *F. mandshurica*, and *T. amurensis* could be planted in the forest management process. At the same time, to end the aggregation of the trees in the middle and lower layers of the forest gaps in advance, non-main tree species could be removed to promote the growth of the main trees. The regeneration advantage of Korean pine in secondary forest plots existed before and after the formation of forest gaps. In secondary forests, Korean pine successfully grew from the lower to the upper layers, whereas it failed to do so in primary forests. The competition differed between tree species in canopy gaps of primary and secondary forests manifested in the medium layer. In secondary forest gaps, shade-tolerant and intermediate species replaced the regeneration advantage of light-demanding trees. In primary forest gaps, the regeneration advantage of intermediate trees were consistently maintained. Our findings further explained the spatial pattern of trees in the gap-filling phase.

## Data availability statement

The original contributions presented in the study are included in the article/**Supplementary Material**. Further inquiries can be directed to the corresponding author.

## Author contributions

DW: Data curation, Formal Analysis, Investigation, Writing - original draft, Writing - review & editing. DL: Data curation, Formal Analysis, Methodology, Writing - original draft, Writing - review & editing. JZ: Conceptualization, Funding acquisition, Methodology, Project administration, Writing - review & editing. XG: Data curation, Investigation, Writing - review & editing. JZ: Data curation, Investigation, Writing - review & editing. LL: Formal analysis, Writing - review & editing. XW: Writing - review & editing. HL: Data curation, Investigation, Writing - review & editing. GZ: Methodology, Writing - review & editing.
