# Supplementary material for: Spatial and temporal regeneration patterns within gaps in the primary forests vs. secondary forests of Northeast China
Source: Front Plant Sci. 2023 Nov 28;14:1305535. doi: 10.3389/fpls.2023.1305535 (PMC10715057; doi:10.3389/fpls.2023.1305535)
Supplement: Supplementary file 1 [file DataSheet_1.docx]

Supplementary Material

# Supplementary Tables and Figures

**Table S1** Importance value (IV) of all species with different levels of shade tolerance. PYG: primary young gap; POG: primary old gap; PCK: primary closed forest; SYG: secondary young gap; SOG: secondary old gap; SCK: secondary closed forest.

| Species | IV (%) | | | | | | Shade tolerance |
| --- | --- | --- | --- | --- | --- | --- | --- |
|  | PYG | POG | PCK | SYG | SOG | SCK |  |
| *Abies holophylla* | 1.0 | 1.3 | 1.4 | 0.8 | 1.5 | 1.3 | shade-tolerant |
| *Abies nephrolepis* | 0.6 | 1.0 | 2.5 | 3.3 | 0.4 | 2.8 | light-demanding |
| *Acanthopanax senticosus* | 5.0 | 3.7 | 7.0 | 1.3 | 1.2 | 1.3 | intermediate |
| *Acer mandshuricum* | 6.4 | 5.6 | 1.8 | 1.4 | 1.4 | 2.2 | shade-tolerant |
| *Acer mono* | 9.3 | 8.0 | 4.1 | 5.8 | 6.6 | 4.0 | intermediate |
| *Acer pseudosieboldianum* | 7.2 | 5.0 | 5.1 | 2.7 | 5.2 | 2.6 | intermediate |
| *Acer tegmentosum* | 9.0 | 8.3 | 5.5 | 5.5 | 4.9 | 2.4 | intermediate |
| *Acer ukurunduense* | 0.5 | 1.7 | 1.0 | 2.3 | 1.4 | 1.1 | shade-tolerant |
| *Albizia kalkora* | 1.0 | 1.7 | 0.5 | 0.9 | 0.9 | 0.5 | intermediate |
| *Aralia elata* | 0.1 | 0.4 | 0.4 | 0.4 | 0.4 | 0.4 | light-demanding |
| *Armeniaca mume* | 9.2 | 5.5 | 7.4 | 3.3 | 3.4 | 3.7 | light-demanding |
| *Betula costata* | 0.1 | 1.5 | 0.0 | 0.1 | 1.4 | 0.9 | light-demanding |
| *Betula platyphylla* | 0.5 | 1.2 | 0.0 | 1.5 | 0.8 | 19.4 | light-demanding |
| *Bothrocaryum controversum* | 0.0 | 0.0 | 0.0 | 0.4 | 0.6 | 0.0 | light-demanding |
| *Buxus Sinica* | 4.2 | 6.2 | 5.1 | 3.3 | 4.8 | 3.6 | light-demanding |
| *Carya cathayensis* | 3.0 | 2.4 | 1.6 | 1.5 | 4.9 | 0.8 | light-demanding |
| *Corylus mandshurica* | 1.4 | 3.5 | 2.8 | 1.1 | 0.8 | 1.4 | light-demanding |
| *Deutzia glabrata* | 9.6 | 8.4 | 4.8 | 5.0 | 5.2 | 4.1 | intermediate |
| *Euonymus alatus* | 0.7 | 0.4 | 0.0 | 3.5 | 3.6 | 3.2 | shade-tolerant |
| *Fraxinus mandshurica* | 1.8 | 2.8 | 1.0 | 3.2 | 3.4 | 4.4 | intermediate |
| *Juglans mandshurica* | 0.0 | 0.0 | 0.0 | 0.1 | 0.4 | 0.0 | light-demanding |
| *Larix gmelinii* | 0.0 | 0.0 | 0.0 | 0.1 | 0.5 | 0.0 | light-demanding |
| *Lonicera japonica* | 0.1 | 1.4 | 1.4 | 0.1 | 0.5 | 0.0 | intermediate |
| *Lycium chinense* | 0.1 | 0.4 | 0.0 | 0.4 | 0.6 | 0.4 | light-demanding |
| *Malus baccata* | 0.5 | 0.4 | 0.5 | 1.2 | 0.9 | 1.3 | light-demanding |
| *Phellodendron amurense* | 0.5 | 0.1 | 0.5 | 0.4 | 1.4 | 0.7 | light-demanding |
| *Philadelphus incanus* | 0.0 | 1.1 | 0.4 | 0.0 | 0.0 | 0.0 | light-demanding |
| *Physalis peruviana* | 2.0 | 0.0 | 2.1 | 1.3 | 1.2 | 1.5 | light-demanding |
| *Pinus koraiensis* | 1.4 | 2.0 | 22.6 | 6.9 | 10.4 | 4.7 | light-demanding |
| *Populus* | 0.1 | 2.3 | 0.4 | 2.1 | 1.6 | 4.1 | light-demanding |
| *Prunus padus* | 3.0 | 3.0 | 0.6 | 1.6 | 1.7 | 1.2 | shade-tolerant |
| *Quercus mongolica* | 1.9 | 3.0 | 1.5 | 2.8 | 3.2 | 1.5 | light-demanding |
| *Rhamnus davurica* | 0.0 | 0.4 | 0.0 | 0.0 | 1.0 | 0.7 | shade-tolerant |
| *Ribes mandshuricum* | 2.0 | 2.3 | 2.3 | 2.2 | 2.5 | 1.9 | light-demanding |
| *Salix* | 0.3 | 0.6 | 0.0 | 4.9 | 0.7 | 0.5 | light-demanding |
| *Sambucus williamsii* | 1.0 | 1.5 | 0.9 | 0.8 | 0.4 | 0.7 | light-demanding |
| *Sorbaria sorbifolia* | 1.0 | 1.4 | 0.9 | 3.8 | 1.6 | 3.1 | light-demanding |
| *Sorbus alnifolia* | 0.6 | 0.4 | 0.0 | 0.4 | 0.0 | 0.4 | light-demanding |
| *Syringa reticulata* | 3.1 | 4.4 | 3.0 | 14.6 | 9.8 | 10.6 | intermediate |
| *Tilia amurensis* | 5.0 | 3.1 | 4.8 | 1.4 | 3.4 | 1.5 | shade-tolerant |
| *Ulmus laciniata* | 2.3 | 2.6 | 2.8 | 0.4 | 0.8 | 0.8 | intermediate |
| *Ulmus pumila* | 3.5 | 0.1 | 2.3 | 6.2 | 3.8 | 3.2 | intermediate |
| *Viburnum dilatatum* | 1.0 | 0.9 | 1.0 | 1.0 | 0.8 | 1.1 | shade-tolerant |

**Table S2** Specie richness in the lower (height: 0~5 m), medium (height: 5~10 m), and upper layer (height: >10 m) in PYG, POG, PCK, SYG, SOG, and SCK.

| Regeneration layer | PYG | POG | PCK | SYG | SOG | SCK |
| --- | --- | --- | --- | --- | --- | --- |
| Lower layer | 30 | 37 | 31 | 36 | 40 | 36 |
| Medium layer | 16 | 23 | 22 | 16 | 14 | 16 |
| Upper layer | 9 | 12 | 16 | 7 | 12 | 19 |
| ALL | 38 | 39 | 33 | 41 | 41 | 38 |

**
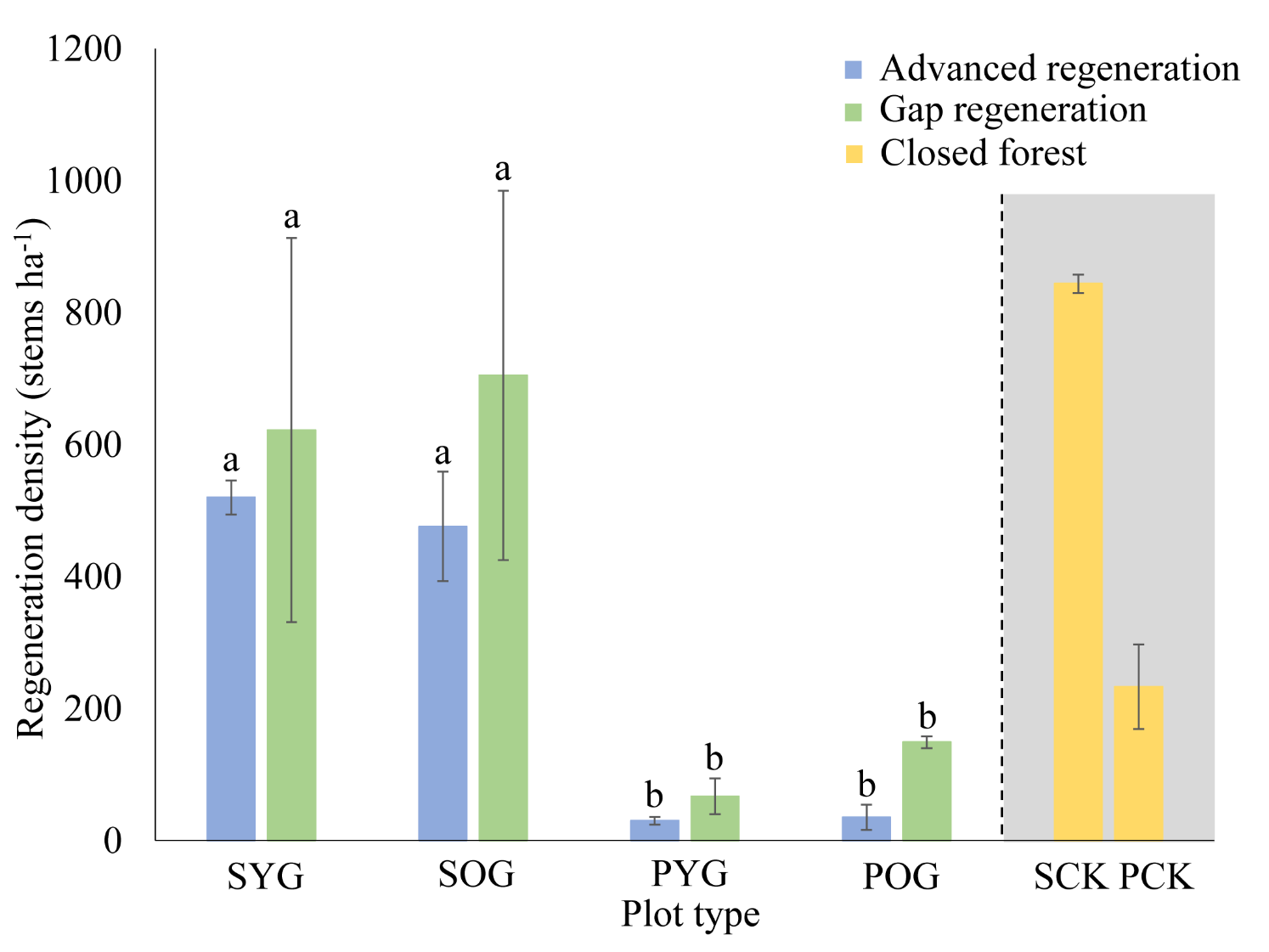
**

**Figure S1** Advanced regeneration and gap regeneration of Korean pine. PYG: primary young gap; POG: primary old gap; PCK: primary closed forest; SYG: secondary young gap; SOG: secondary old gap; SCK: secondary closed forest. Different letters represented significant differences at the *p*<0.05 level.
